# Arachidonic Acid Stress Impacts Pneumococcal Fatty Acid Homeostasis

**DOI:** 10.3389/fmicb.2018.00813

**Published:** 2018-05-11

**Authors:** Bart A. Eijkelkamp, Stephanie L. Begg, Victoria G. Pederick, Claudia Trapetti, Melissa K. Gregory, Jonathan J. Whittall, James C. Paton, Christopher A. McDevitt

**Affiliations:** ^1^Research Centre for Infectious Diseases, School of Biological Sciences, University of Adelaide, Adelaide, SA, Australia; ^2^Adelaide Medical School, University of Adelaide, Adelaide, SA, Australia

**Keywords:** host lipids, free fatty acids, membrane fluidity, macrophages, antibacterial fatty acids, FASII

## Abstract

Free fatty acids hold dual roles during infection, serving to modulate the host immune response while also functioning directly as antimicrobials. Of particular importance are the long chain polyunsaturated fatty acids, which are not commonly found in bacterial organisms, that have been proposed to have antibacterial roles. Arachidonic acid (AA) is a highly abundant long chain polyunsaturated fatty acid and we examined its effect upon *Streptococcus pneumoniae*. Here, we observed that in a murine model of *S. pneumoniae* infection the concentration of AA significantly increases in the blood. The impact of AA stress upon the pathogen was then assessed by a combination of biochemical, biophysical and microbiological assays. *In vitro* bacterial growth and intra-macrophage survival assays revealed that AA has detrimental effects on pneumococcal fitness. Subsequent analyses demonstrated that AA exerts antimicrobial activity via insertion into the pneumococcal membrane, although this did not increase the susceptibility of the bacterium to antibiotic, oxidative or metal ion stress. Transcriptomic profiling showed that AA treatment also resulted in a dramatic down-regulation of the genes involved in fatty acid biosynthesis, in addition to impacts on other metabolic processes, such as carbon-source utilization. Hence, these data reveal that AA has two distinct mechanisms of perturbing the pneumococcal membrane composition. Collectively, this work provides a molecular basis for the antimicrobial contribution of AA to combat pneumococcal infections.

## Introduction

Host-derived free fatty acids play key roles in the defense against pathogenic bacteria, viruses and fungi (Kohn et al., 1980; Zheng et al., 2005; Ells et al., 2009; Desbois and Smith, 2010). Although primarily associated with compromising membrane integrity, studies from a range of different bacterial species have shown that free fatty acids affect other cellular processes, including energy production, nutrient uptake and enzyme activity, in niche-specific and organism-dependent manners (Desbois and Smith, 2010). Free fatty acids have also been shown to impair colonization by pathogenic microbes at the host-pathogen interface (Barakat et al., 2015; Mil-Homens et al., 2016). The broad-spectrum and potential antimicrobial activity of fatty acids has attracted significant interest as possible therapeutic agents (Jackman et al., 2016; Mil-Homens et al., 2016). However, determining the antimicrobial efficacy of free fatty acids is complicated by the fact that many bacterial pathogens have the ability to use host fatty acids as an energy source and/or in the synthesis of their own membranes (Balemans et al., 2010). A prominent example is *Mycobacterium tuberculosis*, wherein persistence within macrophages is believed to be reliant upon incorporation of host fatty acids into the bacterial cell (Peyron et al., 2008; Caire-Brändli et al., 2014). Accordingly, the advantage conferred by use of host fatty acids is likely to be greatest when the host fatty acids are similar to the bacterium's own endogenous fatty acids. Consistent with this inference, host-produced long chain-polyunsaturated fatty acids (LC-PUFAs; ≥20 carbons and ≥2 double bonds) often display greater antibacterial potential than medium/short chain saturated and monounsaturated fatty acids (≥18 carbons and 0 or 1 double bonds), which are produced by both the host and bacteria (Desbois and Smith, 2010). The omega-6 LC-PUFA arachidonic acid (20:4n-6) is known as a precursor for the immunomodulatory prostaglandins (Bhowmick et al., 2013, 2017), but it is also one of the most abundant broad spectrum antimicrobial fatty acids, affecting bacteria, viruses and fungi (Kohn et al., 1980; Zheng et al., 2005; Ells et al., 2009).

*Streptococcus pneumoniae* (the pneumococcus) remains the world's foremost bacterial pathogen, responsible for the deaths of ~1 million individuals annually. Many of its virulence factors have been analyzed for their contribution to disease and survival at the host-pathogen interface. Despite this, there is a relative paucity of knowledge regarding the antimicrobial contribution of fatty acids on the ability of the pneumococcus to cause disease. Pneumococcal fatty acids required for the cell membrane are synthesized by the type II fatty acid synthase (FASII) system (Zhang and Rock, 2008), which is encoded from a single gene cluster (SPD_0378-0390; *fab*-cluster). Regulation of this cluster is mediated by the MarR-type regulator FabT (SPD_0379), which represses transcription of the *fab*-cluster upon binding of fatty acids (Jerga and Rock, 2009). In addition to *de novo* synthesis by the FASII system, the pneumococcus has the ability to incorporate exogenous fatty acids into its membrane through the FakA/B system (Parsons et al., 2015). The components of both the FASII system and FakA are essential for *in vivo* survival (van Opijnen and Camilli, 2012). However, the mechanism(s) through which antimicrobial fatty acids affect the FASII and FakA/B systems, and other crucial cellular processes remains largely unknown. Despite this, studies have shown an association between exogenous fatty acids and restriction of pneumococcal colonization. A recent study of *Corynebacterium accolens* showed that the bacterial triacylglycerol (TAG) lipase could cleave host TAGs generating antimicrobial free fatty acids that impacted pneumococcal colonization (Bomar et al., 2016). This corresponded with *Corynebacterium spp*. being overrepresented in children that were not colonized with *S. pneumoniae*. Further, alveolar macrophages isolated from mice fed on a diet rich in LC-PUFAs exhibited enhanced phagocytic clearance of pneumococci (Saini et al., 2013). These studies highlight the ability of exogenous fatty acids to restrict pneumococcal colonization, although the molecular basis for the antimicrobial activity of free fatty acids remains unknown.

In this study, we examine the antimicrobial potential of arachidonic acid (AA) against *S. pneumoniae* in *in vitro* and cell culture assays. At a molecular level, our data reveal that AA elicits antimicrobial activity against *S. pneumoniae*, altering the pneumococcal membrane via two distinct mechanisms. Our genome-wide analyses did not uncover any resistance mechanisms to counteract AA toxicity, highlighting the potential of antimicrobial fatty acids as a highly effective anti-pneumococcal therapy.

## Materials and methods

### *S. pneumoniae* strains and growth conditions

Opaque phase variants of *S. pneumoniae* strain D39 or its *fakB* (SPD_0646) deletion-replacement derivative were examined in this study. The *fakB* mutant was generated by replacement with an erythromycin resistance cassette, as described previously (Plumptre et al., 2014a) using the oligonucleotides listed in Table S1. Bacteria were routinely grown overnight at 37°C with 5% CO_2_ on Columbia agar supplemented with 5% (vol/vol) horse blood. For subsequent assays, bacteria were grown in a casein-based semisynthetic medium (C+Y with 0.2% glucose) (Lacks and Hotchkiss, 1960) or, for challenge of mice, in serum broth (10% heat-inactivated horse serum [Thermo Fisher Scientific] in nutrient broth [Oxoid]). For growth analyses, *S. pneumoniae* D39 and the *fakB* mutant strain were grown in C+Y until they reached an optical density at 600 nm (OD_600_) of 0.3. They were then subcultured into 200 μl C+Y (with or without AA [Sigma-Aldrich], H_2_O_2_, paraquat, gentamicin, chloramphenicol, zinc and/or copper) to a final OD_600_ of 0.01. The bacteria were incubated at 37°C in a CO_2_-enriched atmosphere, with growth monitored by measurement of the OD_600_ at 30 min intervals on a FLUOstar Omega (BMG Labtech).

### Population viability assessment

Following growth to mid-log phase in C+Y medium at 37°C with 5% CO_2_, cells were washed three times to remove excess AA. Cells were then shock-treated with 4 μg.mL^−1^ LL-37 for 30 min in PBS. The viability of washed cells was determined by staining for 5 min with Sytox Green (Molecular Probes). At least 8,000 cells were then assessed on a BD Accuri flow cytometer, with the data analyzed using FlowJo 10.2 (BD). Dead cells (Sytox Green positive) were gated using the FITC intensity of unlabeled cells. AA treatment alone did not result in increased cell death of D39, with only LL-37 inducing death. Data represent an average of at least biological triplicates.

### Murine infection

Murine infection experiments were conducted as described previously (Plumptre et al., 2014a,b; Eijkelkamp et al., 2016). Outbred 5- to 6-week old female CD1 (Swiss) mice were used in all animal experiments. Mice were anesthetized by intraperitoneal injection of pentobarbital sodium (Nembutal; Rhone-Merieux) at a dose of 66 μg.g of body weight^−1^, followed by intranasal administration of 50 μl bacterial suspension in serum broth, containing approximately 1 × 10^7^ colony forming units (CFUs). The challenge dose was confirmed retrospectively by serial dilution and plating on blood agar. At 24 h post-challenge, mice were euthanized by CO_2_ asphyxiation. Blood was collected by syringe from the posterior vena cava and transferred to a heparinized tube. A small aliquot was serially diluted and plated on blood agar for CFU counts. Plasma was isolated from the remainder of the sample and submitted for fatty acid analyses as described below. All procedures performed in this study were conducted with a view to minimizing the discomfort of the animals, and used the minimum numbers to generate reproducible and statistically significant data. All experiments were approved by the University of Adelaide Animal Ethics Committee (Animal Welfare Assurance number A5491-01; project approval number S-2013-053) and were performed in strict adherence to guidelines dictated by the Australian Code of Practice for the Care and Use of Animals for Scientific Purposes.

### Fatty acid analyses

For analysis of the bacterial fatty acids, cultures were grown to an OD_600_ of 0.3 in C+Y media at 37°C with 5% CO_2_, cells were washed in phosphate buffered saline (PBS) and then disrupted by sonication for 60 cycles (30 s on and 30 s off) on a Bioruptor (Diagenode). The fatty acid content of the bacterial cell lysates and murine plasma was then analyzed by GC-MS at the Waite Analytical Services (University of Adelaide) as described previously (Eijkelkamp et al., 2013; Pederick et al., 2014). The statistical differences between samples (*n* ≥ 3) were examined using an unpaired Student *t*-test (Graphpad Prism 6.0c).

### Macrophage killing assays

Macrophage killing assays were performed as previously described (Hassan et al., 2017; Martin et al., 2017). THP-1 cells (ATCC TIB-202) were grown under atmospheric control (95% air and 5% CO_2_) at 37°C in complete RPMI medium (RPMI with phenol red [Gibco], supplemented with 10% fetal bovine serum, 10 mM HEPES, 30 μg.mL^−1^ penicillin and 50 μg.mL^−1^ streptomycin). Cell culture flasks (25 cm^2^; BD Falcon) were seeded with 3.5 × 10^6^ THP-1 cells and differentiated by adding 100 ng.mL^−1^ phorbol 12-myristate 13-acetate (PMA; Sigma-Aldrich) and incubated for 3 days. Attached differentiated THP-1 cells (macrophages) were washed in complete RPMI and incubated with complete RPMI without added PMA for > 2 days to allow resting. Two hours before challenge with *S. pneumoniae*, a subset of macrophages was treated with 5 μM AA (Sigma-Aldrich), which represents a concentration appropriate for treatment of cell cultures (Caruso et al., 1994; Ghioni et al., 1997; Hughes-Fulford et al., 2005; Gregory et al., 2011). The AA was pre-incubated with fatty acid free BSA (Sigma) at a 4:1 ratio (AA:BSA) to allow for efficient delivery of AA to THP-1 cells, following standard fatty acid supplementation procedures (Ghioni et al., 1997). Following thorough washing (3 times in Hank's Balanced Salt Solution [HBSS; Thermo Fisher Scientific]), THP-1 cells were detached using 1 mL StemPro Accutase (Thermo Fisher Scientific) and washed again for 3 times to remove the residual StemPro Accutase. Following viability assessment and enumeration by trypan blue staining and microscopy, THP-1 cells were diluted to 1.1 × 10^5^ cells.mL^−1^ in HBSS.

*S. pneumoniae* D39 were grown overnight on blood agar plates at 37°C with 95% air and 5% CO_2_ and subsequently inoculated into C+Y media (Begg et al., 2015) to an OD_600_ of 0.05. The cultures were grown at 37°C with 5% CO_2_ until the OD_600_ reached 0.3, after which the cells were washed, resuspended in HBSS, and CFU counts determined by plating on blood agar. The macrophages and *S. pneumoniae* cells were co-incubated at a ratio of 1:10 for 90 min. The macrophages were then washed and extracellular bacteria were killed by incubation with 200 μg.mL^−1^ gentamicin and 10 μg.mL^−1^ penicillin for 30 min. The macrophages were washed in HBSS without antibiotic and incubated for a further 60 min prior to analysis of intracellular bacteria by lysing the macrophages with 0.0625% Triton-X-100. The lysates were then plated onto blood agar. The CFUs were enumerated and corrected for input. The data are the mean of at least four biological replicates (±SEM). The statistical differences between pneumococcal survival in AA-treated and untreated THP-1 cells were examined using an unpaired Student *t*-test.

### qRT-PCR analyses

For isolation of RNA, 500 μl of a *S. pneumoniae* culture at an OD_600_ of 0.3, grown in C+Y media at 37°C with 5% CO_2_, was mixed with 1 ml of RNA Protect (Qiagen). RNA was extracted and purified using an RNeasy Bacteria Mini Kit (Qiagen) after enzymatic lysis using lysozyme and mutanolysin, as described previously (Eijkelkamp et al., 2014a, 2015, 2016; Plumptre et al., 2014a,b; Begg et al., 2015). The total RNA samples were treated with DNase I (Roche) and qRT-PCR was carried out using a SuperScript III One-Step RT-PCR kit (Thermo Fisher Scientific) on a LC480 Real-Time Cycler (Roche). Transcription levels of genes analyzed were normalized to those obtained for 16S rRNA. Primer sequences are available in Table S1. Results are representative of at least four independent samples and the statistical difference was examined by an unpaired Student *t*-test (Graphpad Prism 6.0c).

### Transcriptomic analyses

RNA isolated from biological quadruplicates of *S. pneumoniae* D39 grown with or without 62.5 μM AA in C+Y media at 37°C with 5% CO_2_ was pooled and submitted to the Australian Genomics Research Facility for sequencing. Briefly, the RiboZero bacterial rRNA removal kit (Illumina) was used to reduce the ribosomal RNA content of the total RNA pool, followed by use of the TruSeq Stranded mRNA Library Prep Kit (Illumina) to generate the barcoded libraries. Prepared libraries were then sequenced using the Illumina HiSeq2500 with Version 3 SBS reagents and 1 × 100 bp single-end chemistry. Reads were aligned to the *S. pneumoniae* D39 genome using BOWTIE2 version 2.2.3 (Langmead and Salzberg, 2012). Counts for each gene were obtained with the aid of SAMtools (v 0.1.18) (Li et al., 2009) and BEDtools (Quinlan and Hall, 2010) and differential gene expression was examined using R DESeq (Anders and Huber, 2010; Pederick et al., 2015). In the text and in **Figure 4** and Table S2 we have highlighted the genes that were found to be statistically different as determined by DESeq. The complete data set has been submitted to GEO (accession number GSE93102).

### Membrane fluidity

Bacterial cultures were grown to an OD_600_ of 0.3 in C+Y media at 37°C with 5% CO_2_ and cells were washed in PBS. Bacteria were than incubated with 1,6-diphenyl-1,3,5-hexatriene (DPH), which was dissolved in tetrahydrofuran. After incubation at 37°C for 30 min, cells were washed and fluorescence polarization (excitation 350/emission 450 nm) was determined on a PHERAstar spectrophotometer (BMG Labtech). The relative change in membrane fluidity was determined from at least 3 independent experiments and the statistical difference was assessed by an unpaired Student *t*-test.

### Whole cell metal ion accumulation

*S. pneumoniae* strains were grown to an OD_600_ of 0.3 in C+Y media at 37°C with 5% CO_2_ and the total cell-associated metal ion content was determined essentially as described previously (Eijkelkamp et al., 2014a, 2016; Pederick et al., 2015). Succinctly, bacteria were washed three times with PBS + 5 mM EDTA, then washed four times with PBS. Bacterial pellets were desiccated by heating at 95°C overnight. The dry cell weight was measured and the pellets resuspended in 35% HNO_3_. Metal ion content was measured on an Agilent 7500cx inductively coupled plasma-mass spectrometer (Adelaide Microscopy). The data represents 6 biological replicates and the statistical difference assessed by an unpaired Student *t*-test (Graphpad Prism 6.0c).

### Ethanol detection assays

Intracellular ethanol abundance was determined using Megazyme Ethanol Detection kit as per manufacturer's instructions. Briefly *S. pneumoniae* D39 was grown in C+Y with or without 62.5 μM AA supplementation, to an OD_600_ of 0.3 at 37°C with 5% CO_2_. One milliliter of culture was harvested via centrifugation (12,000 × *g* for 5 min) and resuspended in 200 μl of ethanol-free ddH_2_O. Bacterial cells were lysed by incubation with 0.1% (v/v) deoxycholate for 10 min at 37°C, and 100 μl of cell lysate was analyzed for ethanol abundance via absorbance at 340 nm on a PHERAstar spectrophotometer (BMG Labtech). The data represents > 4 biological replicates and statistical analyses were performed by an unpaired Student *t*-test (Graphpad Prism 6.0c).

### YtrA and FabT binding site identification

The YtrA and FabT motifs were generated as described previously (Eijkelkamp et al., 2011a,b, 2014b; Giles et al., 2015). For YtrA, the independent binding sites that served as a template were obtained from Suvorova et al. (2015), and for FabT from Marrakchi et al. (2002). In brief, the binding site sequences were aligned using Clustal Omega (Sievers et al., 2011) to generate the binding site motif. The putative binding sites were then identified using HMMER 2.0 (Finn et al., 2011) as an integral part of UGENE (Okonechnikov et al., 2012).

## Results and discussion

### Abundance of arachidonic acid and its antimicrobial activity

**Figure 1:**
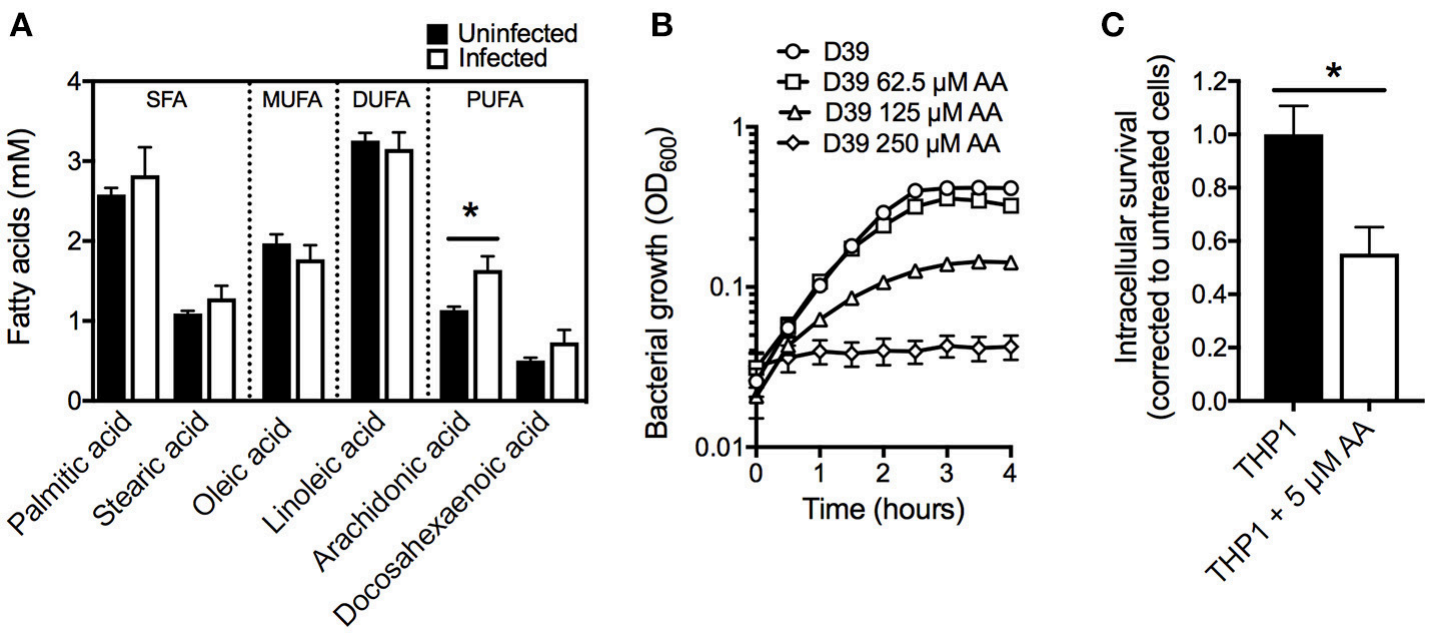
Examination of arachidonic acid as an anti-pneumococcal agent. **(A)** Examination of plasma fatty acids before and after infection with *S. pneumoniae* strain D39. **(B)** Examination of pneumococcal growth upon treatment with AA at 62.5, 125, or 250 μM. The OD_600_ was determined every 30 min. **(C)** Intracellular survival of pneumococci within THP-1 derived macrophages treated with 5 μM AA by comparison with untreated macrophages. All data are the mean of at least biological triplicates (±SEM) and where error bars are not visible this is due to occlusion by the shown symbols. Statistical analyses were performed using a Student's *t*-test (^*^p < 0.05).

To study the role of free fatty acids in restricting pneumococcal proliferation during infection, we first determined the concentrations of plasma fatty acids and their potential fluxes upon infection. Outbred female Swiss mice were intranasally challenged with the virulent *S. pneumoniae* type 2 strain D39 or with PBS as a control. At 24 h post-challenge, the pneumococci caused a systemic infection with an average bacterial count of 1.5 × 10^7^ CFU.mL^−1^ (SD ± 2.3 × 10^6^) in the blood. Plasma fatty acid content of infected, and naïve control mice, were quantitatively determined after methanol-chloroform extraction by gas-chromatography (GC) (Eijkelkamp et al., 2013; Pederick et al., 2014). The acyl chains of the major fatty acids (>1% relative abundance) identified in the plasma were between 16 and 22 carbons in length (Figure 1A). These included two saturated fatty acids, palmitic acid (16:0) and stearic acid (18:0), two omega-6 fatty acids, linoleic acid (18:2n-6) and arachidonic acid (20:4n-6), the omega-9 fatty acid oleic acid (18:1n-9) and the omega-3 fatty acid docosahexaeonic acid (22:6n-3). Significant changes in AA were observed upon the establishment of infection, from 1.1 to 1.6 mM (44% increase; *p* < 0.05), in our pneumonia-bacteremia model of pneumococcal infection. This specific modulation of AA abundance in plasma suggests that it may play an important role in host defense against pneumococcal infection. AA is primarily known for its pro-inflammatory activity, serving as a major precursor for classic eicosanoids, such as prostaglandins, although this fatty acid has also been associated with antimicrobial activity against a range of pathogenic organisms (Kohn et al., 1980; Das, 1985; Ells et al., 2009; Desbois and Smith, 2010; Jackman et al., 2016; Mil-Homens et al., 2016). Here we focused on the direct antimicrobial properties of this LC-PUFA and sought to examine this *in vitro*. We observed that *S. pneumoniae* D39 is highly susceptible to AA, with growth significantly perturbed upon supplementation with 62.5 μM AA into the culture medium. Growth of the pathogen was almost completely abrogated by supplementation with 125 μM AA (Figure 1B). Notably, the concentrations examined here are far below those observed in murine plasma (1.6 mM; Figure 1A), suggesting a physiological relevance of potent AA toxicity during infection.

Fatty acids play important roles in phagocytic cells, where omega-6 fatty acids, such as AA, are associated with enhanced phagocytic functionality by mechanisms such as improved phagolysosome maturation and greater bacterial killing (Anes et al., 2003; Jordao et al., 2008). Further, the release of AA from membrane phospholipids is critical for the production of the neutrophil chemoattractant, hepoxillin A_3_, which has key roles in systemic pneumococcal infections (Bhowmick et al., 2017). Fatty acids have also been proposed to target phagocytosed pathogens within macrophages through fusion of the phagolysosome with lipid bodies containing high concentrations of fatty acids (Adolph et al., 2012; Caire-Brändli et al., 2014). Given the observed susceptibility of *S. pneumoniae* to AA and the significant role of AA in immune modulation, we hypothesized that AA may play a crucial role in controlling pneumococci within macrophages. Here, we ascertained the effect of AA on human THP-1 derived macrophages, by examining loading of *S. pneumoniae* within AA-treated macrophages compared to untreated macrophages. We observed nearly a 50% reduction in pneumococcal loading by AA-treated macrophages by comparison with untreated macrophages (Figure 1C), highlighting the importance of AA in phagocytic cells. The reduction in pneumococcal loading in AA-treated macrophages may arise from AA improving lysosomal maturation or by AA interfering with the internalization of pneumococci. Alternatively, AA may have a direct antimicrobial role within macrophages and enhance killing of the phagocytosed bacteria. Irrespective of the precise mechanism, our analyses of serum AA levels during infection, the effect of AA on macrophages and the direct impact of AA on pneumococcal fitness, shows the significance of AA in combatting pneumococcal infections.

### Arachidonic acid targets the pneumococcal membrane

**Figure 2:**
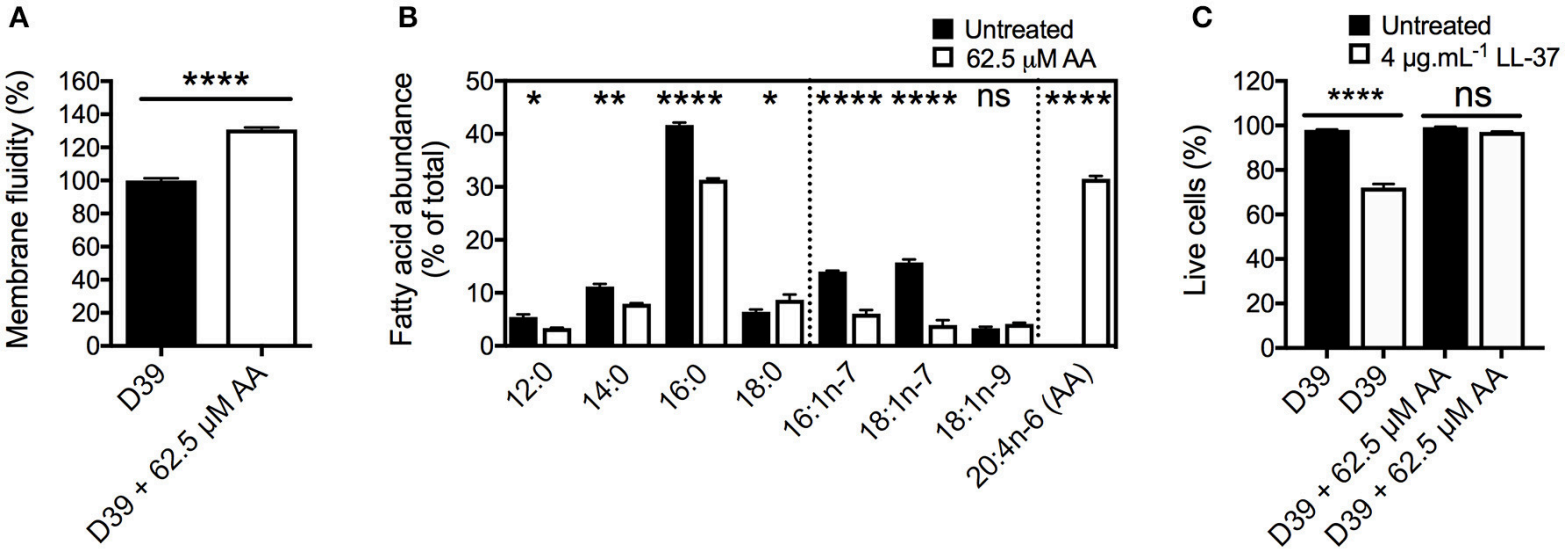
The effects of arachidonic acid on the pneumococcal membrane. **(A)** The membrane fluidity was determined by examining the fluorescence polarization of 1,6-diphenyl-1,3,5-hexatriene (DPH) in the cellular membrane. The relative membrane fluidity in AA-treated cells (62.5 μM) was corrected to that observed in untreated cells. **(B)** The major cellular fatty acid constituents of *S. pneumoniae*, grown in the presence of 62.5 μM AA, or without AA supplementation were determined by gas chromatography. The abundance of fatty acids is expressed as percentage of total cellular fatty acids. **(C)** The survival following shock-treatment with 4 μg.mL^−1^ LL-37 for 30 min was determined by staining with Sytox green (Molecular Probes) and subsequent flow cytometric cell counting (BD Accuri). For all panels, the data are the mean of at least biological triplicates (± SEM). For the membrane fluidity **(A)** and fatty acid analyses **(B)**, statistical analyses were performed by Student's *t*-test (ns, not significant; ^*^p < 0.05; ^**^p < 0.01, and ^****^p < 0.0001). For the cell viability assay **(C)**, statistical analyses were performed using a one-way ANOVA (ns, not significant; ^****^p < 0.0001).

One route by which AA exerts an impact on bacterial organisms is via membrane incorporation. Here, we sought to investigate whether this occurred in *S. pneumoniae* by examining cells grown in the presence of 62.5 μM AA and assessing membrane fluidity by fluorescence polarization using 1,6-diphenyl-1,3,5-hexatriene (DPH). These assays showed that pneumococcal membranes were significantly impacted by this relatively mild treatment (15-min growth delay at mid-log phase), with membrane fluidity increased by 31% (*p* < 0.0001) compared to untreated cells (Figure 2A).

Membrane fluidity is predominantly determined by the acyl groups of the fatty acids. To examine the effects of AA treatment on the pneumococcal cell membrane composition, we quantitatively determined the fatty acid content of untreated and 62.5 μM AA treated cells (Figure 2B). In untreated cells, we identified 4 saturated fatty acids, 12:0 (5.4%), 14:0 (11.1%), 16:0 (41.6%), and 18:0 (6.4%), as well as 3 monounsaturated fatty acids; 16:1n-7 (14.0%), 18:1n-7 (15.7%), and 18:1n-9 (3.3%). Upon treatment, we observed that AA was readily incorporated into the cell, resulting in AA abundance accounting for 31.5% of the cellular fatty acids (Figure 2B). Taken together, these data provide a plausible basis for the observed increase in the membrane fluidity in AA-treated cells.

Intriguingly, the impact of AA treatment upon the endogenous fatty acid content of the pneumococcal membrane was greatest for the 2 monounsaturated omega-7 fatty acids, 16:1n-7 and 18:1n-7, which decreased by more than 2- and 3-fold, respectively (Figure 2B). Although relatively less dramatic, the abundance of 16:0 decreased by approximately 10%. Minor decreases were observed in the abundance of 12:0 and 14:0, whereas 18:0 showed a small increase.

To examine the effect of the changes in the membrane composition and its fluidity on stress resistance, we examined the survival of untreated and AA-treated cells to the human antimicrobial peptide LL-37 (Figure 2C). First, the FSC-SSC profiles of pneumococci were assessed and observed to not be significantly affected by treatment with AA, LL-37 or the combination of these antimicrobials. Interestingly, AA-treated cells were highly resistant to LL-37, with a 72% survival in untreated cells and 97% in AA-treated cells (*p* < 0.0001). This may be a result of AA binding the membrane targeting peptide, thereby diminishing its antimicrobial potential. Treatment with AA did not significantly change pneumococcal growth in the presence of the hydrophilic antibiotic gentamicin or the hydrophobic antibiotic chloramphenicol (Figure S1A,B). Although AA and gentamicin exert an additive antimicrobial effect, treatment with AA and chloramphenicol elicited the same effect as treatment with chloramphenicol only. Metal ion intoxication by copper and zinc is known to exert potent antimicrobial activity toward *S. pneumoniae* (Eijkelkamp et al., 2014a; Johnson et al., 2015) and this has been shown to act synergistically with AA in other bacteria (Hassan et al., 2017). However, treatment of pneumococci with AA did not reveal major changes in their ability to grow in the presence of zinc or copper. Similar to our observations for chloramphenicol, AA stress did not cause a greater growth perturbation than that observed for the metal stress independently, thereby indicating that altered membrane fluidity did not affect zinc or copper homeostasis (Figure S1C,D).

Collectively, these analyses show that AA stress on *S. pneumoniae* perturbs pneumococcal cell integrity by direct incorporation into the bacterial cell membrane, resulting in altered membrane composition and increased fluidity. Despite this, perturbation of the cell membrane did not increase the susceptibility of the pneumococcus to exogenous antibiotic or metal ion stress. Further, AA treatment provided protection against the membrane-targeting host antimicrobial peptide LL-37.

### Arachidonic acid stress and the effect on the pneumococcal transcriptome

**Figure 3:**
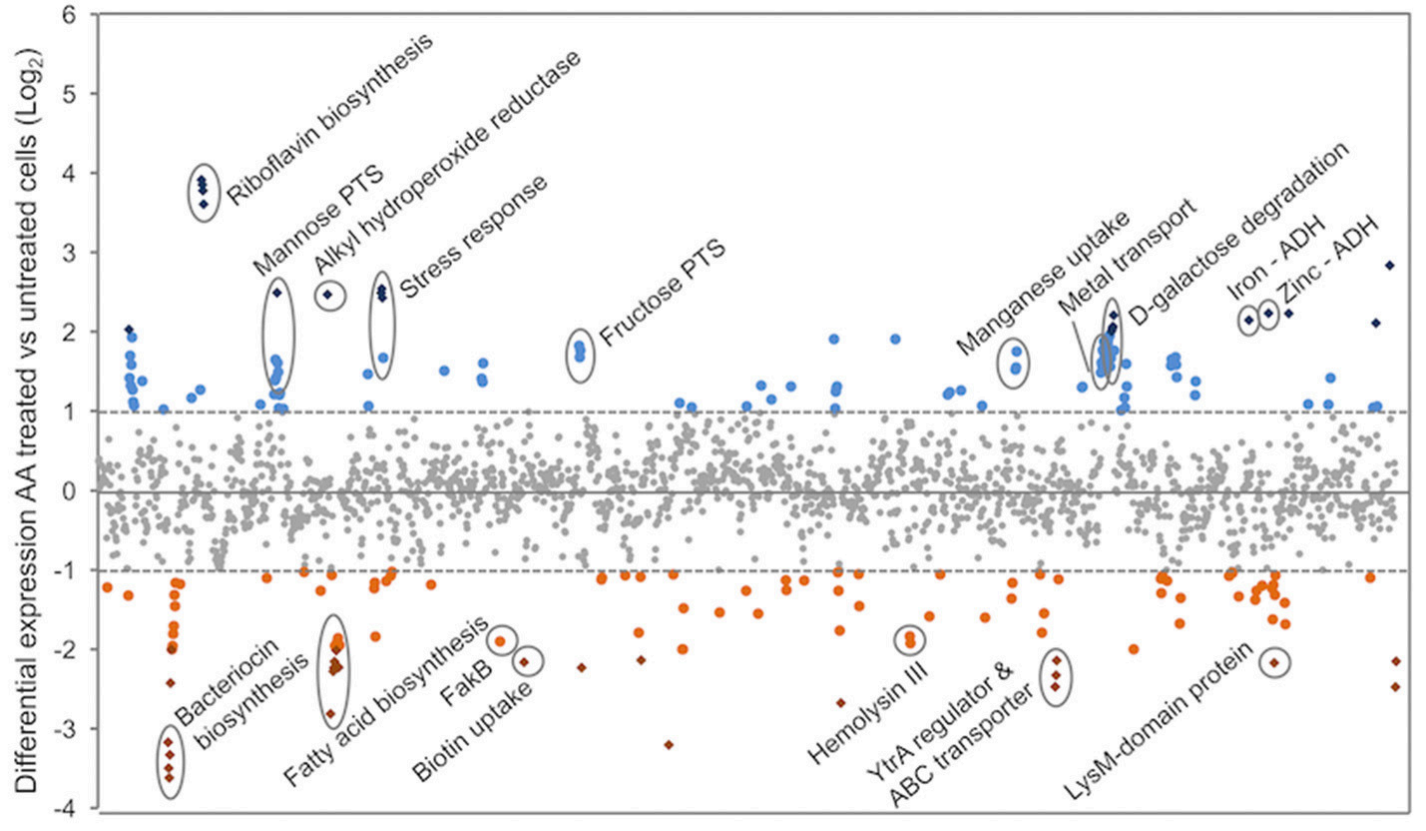
The transcriptomic responses of *S. pneumoniae* to AA treatment. RNA sequencing of untreated and AA-treated (62.5 μM) *S. pneumoniae* D39 was used to determine relative gene expression (expressed as log_2_-fold change compared to untreated). Each symbol represents a gene, with each gene distributed on the X-axis in accordance with locus tag numbering for strain D39. Genes upregulated upon AA treatment are presented above the X-axis (blue circles > 2-fold and blue diamonds > 4-fold), with those below the X-axis expressed at a lower level upon AA treatment (orange circles > 2-fold and orange diamonds > 4-fold). Genes of interest are annotated with their putative or characterized functions.

To gain greater insight into the effect of AA on pneumococcal physiology we examined the complete transcriptomic response of the bacterium to exogenous AA treatment. Pneumococcal cells were grown in the presence of 62.5 μM AA until mid-log phase, with the transcriptomic responses of the two groups (4 pooled biological replicates per condition) examined by RNA sequencing. Overall, 110 genes (5.3%) were up-regulated and 103 genes (5.2%) down-regulated by more than a 2-fold (Figure 3 and Table S2). Further analysis of a subset of 5 genes displayed a strong correlation between analysis by RNA sequencing and qRT-PCR (*R*^2^ = 0.9847; Figure S2).

**Figure 4:**
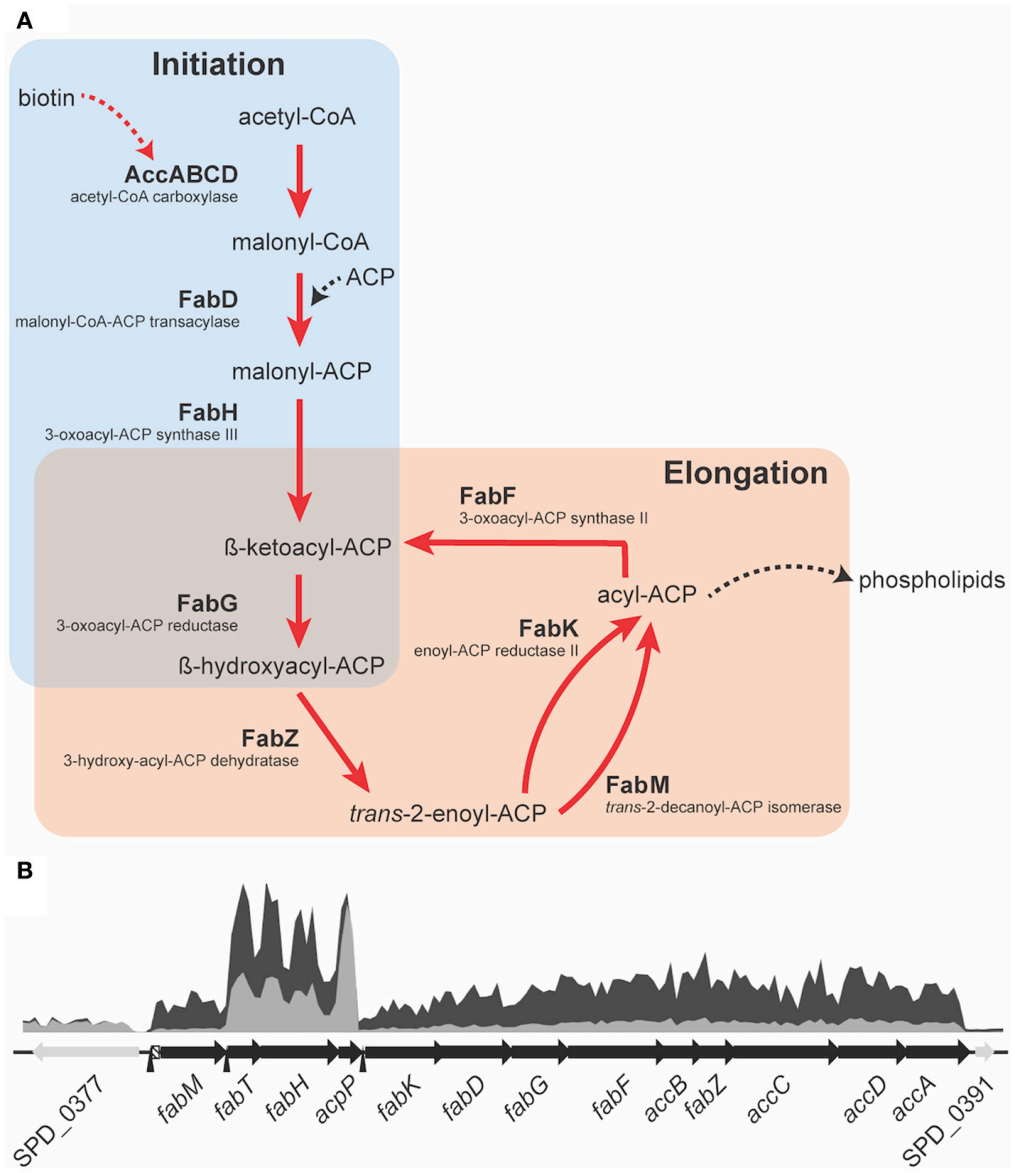
A schematic representation of the fatty acid biosynthesis cluster. **(A)** The fatty acid biosynthesis pathway of the pneumococcus consists of two major parts; initiation (light blue) and elongation (light red). A red arrow indicates genes that are down-regulated upon AA treatment and in black those not affected by AA. ACP is acyl carrier protein and CoA is Coenzyme A. **(B)** The RNAseq coverage of untreated cells (dark gray) and of AA-treated cells (light gray) are presented relative to their genomic positioning. The open reading frames of the components of the *fab*-cluster are indicated, to scale, in the black arrows, and adjacent genes of unrelated function in gray. The putative FabT binding sites are indicated by the black arrow heads, upstream of *fabM, fabT*, and *fabK*. The region of significant strain-to-strain variation upstream of *fabM* is indicated in the box with diagonal stripes.

The *fab* fatty acid biosynthesis cluster was found to be significantly affected by AA (Figure 4A,B). Here, we observed significant down-regulation of the *fab* fatty acid biosynthesis genes ranging from 3.7-fold (*accA*) to 4.8-fold (*fabK*). These findings implicate FabT as being responsive to the increased abundance of AA and mediating repression of the *fab* gene cluster. Prior work has shown that deletion of *fabT* results in increased expression of all genes in the *fab* cluster, except for *fabM* (Lu and Rock, 2006; Jerga and Rock, 2009), despite the presence of a putative FabT binding site upstream of *fabM* (Marrakchi et al., 2002) (Figure 4). Here, we observed that, similar to the other genes in the *fab*-cluster, *fabM* was significantly down-regulated upon exposure to AA (7-fold). Jerga and Rock (2009) showed that FabT had the greatest affinity for the FabT-binding site of *fabK* when bound to the longest unsaturated endogenous fatty acid analyzed in their study, 18:1. By contrast, shorter chain and saturated fatty acids resulted in lower affinity interactions of FabT with the *fabK* operator site (Jerga and Rock, 2009). Hence, we propose that AA (20:4), a LC-PUFA, binds to FabT resulting in a complex that has an increased affinity for its DNA targets, including the putative FabT-binding site upstream of *fabM*. However, we have no direct evidence for this and so an alternative possibility is that endogenous long chain fatty acids may also accumulate in the cytosol leading to highly repressive FabT-complexes. Irrespective of the precise mechanism, whether direct or indirect, the impact on endogenous fatty acid biosynthesis is mediated in response to increased AA abundance.

Despite the fact that *fab* gene clusters show a high level of homology within the species, comparative genomic analyses revealed significant strain-to-strain variation in the genomic region between the FabT-binding site and the start of the *fabM* open reading frame (Figure 4B). This suggests that *fabM* expression may differ between *S. pneumoniae* strains leading to differences in the relative abundance of unsaturated fatty acids and hence membrane fluidity, although further studies will be required to determine veracity of this inference. Transcription of *fabT*, which auto-regulates its expression, and *fabH*, which is co-transcribed with *fabT*, showed minor down-regulation upon AA treatment, a common observation for auto-regulated genes (Eijkelkamp et al., 2011b; Plumptre et al., 2014a). By contrast, transcription of *acpP*, which encodes the acyl-carrier protein (ACP), was not affected by AA stress (Figure 4B), indicating that it is the enzymatic conversions within the FASII pathway that are rate limiting (Figure 4A).

Analysis of pneumococcal fatty acid composition revealed that AA treatment induced the greatest changes on the omega-7 monounsaturated fatty acids (Figure 2B). Down-regulation of *fabM* under AA stress, which plays a key role in the formation of unsaturated fatty acids in the pneumococcal membrane (Lu and Rock, 2006; Figure 4A), could be the cause of the observed depletion of 16:1n-7 and 18:1n-7 in *S. pneumoniae*. In addition to the impacts on fatty acid biosynthesis via the *fab*-gene cluster, other membrane homeostasis-related genes also exhibited differential transcription in response to AA treatment. Transcriptomic analyses revealed that *bioY*, which encodes the biotin uptake protein, was down-regulated by 4.5-fold in response to AA treatment. Biotin is essential for the biosynthesis of fatty acids (Figure 4A) and this finding suggests that during AA stress, the decreased production of endogenous fatty acids may be due a concomitant reduction in biotin accumulation. Disruption of membrane homeostasis is also implicated by the down-regulation of SPD_0646 (3.7-fold), a DegV-like protein suggested to be involved in the maintenance of the cell's fatty acid composition. In *Staphylococcus aureus*, the DegV-like proteins, FakB1 and FakB2, have been shown to be involved in the acquisition of extracellular fatty acids. These fatty acids can subsequently be incorporated in the bacterium's membrane via PlsX (Parsons et al., 2014). Consequently, we investigated the contribution of the putative FakB (SPD_0646) in pneumococcal susceptibility to AA. We observed that the growth of a *fakB* mutant upon treatment with AA was no different to the wild-type (Figure S3). However, it is plausible that *fakB* is dysregulated by AA, with fatty acids other than AA being substrates of the encoded FakB protein. Hence, we examined the susceptibility of the *fakB* mutant to the second most highly abundant LC-PUFA in mouse serum, the omega-3 fatty acid, docosahexaenoic acid (DHA) (Figure 1A). Interestingly, these analyses revealed a minor, but discernable increase in perturbation by 62.5 μM DHA in the *fakB* mutant, by comparison to the wild-type (Figure S3). Therefore, while it is possible that the down-regulation of SPD_0646 is a mechanism to limit AA toxicity, the physiological substrates of the pneumococcal FakB protein are likely to be a range of different types of fatty acids, such as DHA.

SPD_1874, which encodes a putative peptidoglycan-binding LysM-domain containing protein, was also significantly downregulated (4.5-fold) upon AA treatment. The cluster harboring this gene (SPD_1871-76) has previously been shown to be regulated by YycFG, a global two-component regulator (Ng et al., 2003). YycFG has also been shown to regulate *fabT*, with overexpression of YycF resulting in an altered cellular fatty acid composition (Mohedano et al., 2005, 2016). As a consequence, we cannot exclude the possibility that *fabM* may be co-regulated by other systems such as YycFG. Although, not all previously identified regulatory targets of YycFG were found to be differentially expressed upon AA treatment in our study, the SPD_0771-73 gene cluster, which encodes a fructose phosphotransferase system (PTS) repressor (SPD_0771), phosphofructokinase (SPD_0772) and fructose PTS system enzyme II ABC (SPD_0773), was significantly upregulated (Table S2), corroborating previous studies of YycFG (Ng et al., 2003). Transcription of the *yycF* and *yycG* genes increased by 1.3- and 1.2-fold upon AA stress, respectively.

The riboflavin biosynthesis pathway (SPD_0166-9) was the most responsive to AA stress (12- to 15-fold up-regulation). Products derived from riboflavin biosynthesis include the riboflavin derivatives flavin mononucleotide (FMN) and flavin adenine dinucleotide (FAD), which contribute to fatty acid oxidation and oxidative stress resistance (Ross and Hansen, 1992; Ashoori and Saedisomeolia, 2014). Despite this, *ribF* (SPD_0994), which is responsible for the conversion of riboflavin to FMN and FAD was not differentially expressed, indicating that AA-treated pneumococci likely overproduce riboflavin without full conversion to FMN and FAD cofactors. This corroborates previous studies on *Bacillus subtilis*, which have shown that FMN functions as a repressor of the riboflavin biosynthetic pathway (Mack et al., 1998). Although relatively little is known about the function of riboflavin, the vitamin has been shown to directly bind metal ions (Albert, 1950) and as such may serve as a component of the intracellular buffer for metal ions. Pneumococcal metal ion homeostasis is affected by AA stress, with a putative ferric iron ABC permease (SPD_1607-10; ~3.7-fold), a putative magnesium importer (SPD_1606; 3-fold) and the manganese ABC permease (*psaBCA*; ~3-fold; McDevitt et al., 2011; Counago et al., 2014; Eijkelkamp et al., 2014a, 2015) all showing significant up-regulation. Although whole cell metal ion determination did not reveal significant difference in the concentration of first row transition metal ions (manganese iron, copper, or zinc; Figure S4), the increased transcription of these transporters suggest that the intracellular abundance of metal ions sensed by key regulators, such as PsaR, may be perturbed by the increased riboflavin levels.

In addition to the perturbation of metal ion homeostatic regulation, increased transcription of alkyl hydroperoxide reductase (*ahpD*; SPD_0373; 5.5-fold) was observed, suggesting that AA induces oxidative stress (Schönfeld and Wojtczak, 2008; Desbois and Smith, 2010). However, treatment with AA did not result in increased susceptibility of *S. pneumoniae* to H_2_O_2_ or the superoxide-inducing compound, paraquat (Figure S5A,B). As a consequence, the apparent lack of heightened susceptibility may be due to the induction of AhpD and other related pathways in response to AA.

**Figure 5:**
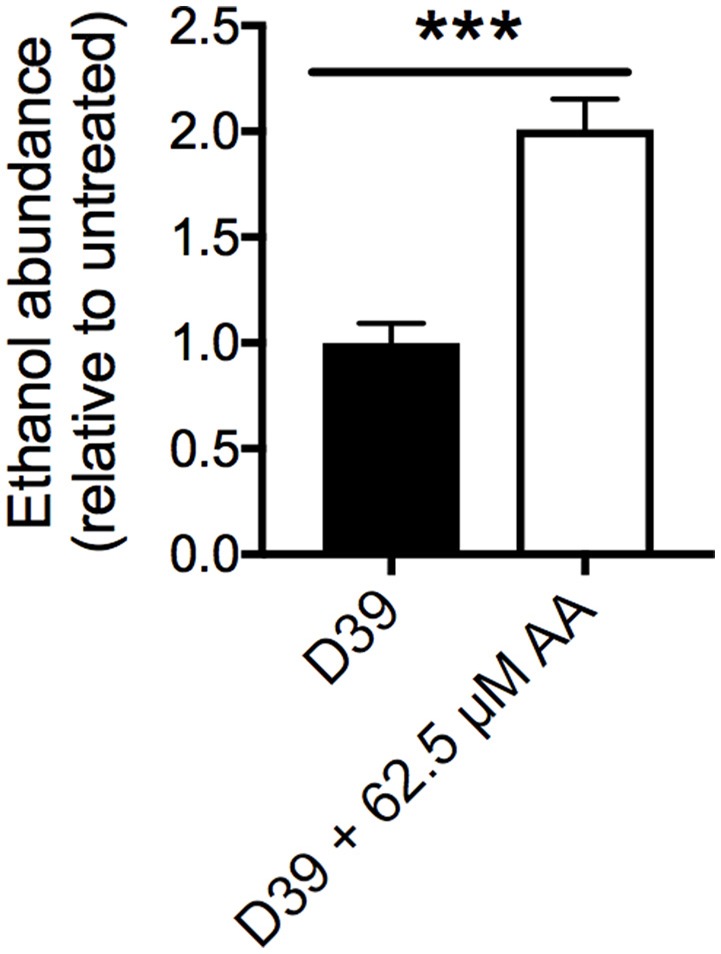
Cellular ethanol abundance. Intracellular ethanol abundance of *S. pneumoniae* D39 grown in the presence (white bar) and absence (black bar) of 62.5 μM AA supplementation. Ethanol abundance is relative to untreated D39. The data are the mean of 5 biological replicates (±SEM). Statistical analyses were performed by Student's unpaired *t*-test (^***^p < 0.0005).

Treatment with AA also resulted in the up-regulation of multiple alcohol dehydrogenases (ADHs) including SPD_1636 (3.0-fold), SPD_1834 (4.4-fold) and SPD_1865 (4.7-fold). To examine the basis for this, the cellular ethanol abundance of untreated and AA-treated cells was assessed, revealing 2-fold higher ethanol accumulation in AA-treated cells by comparison with the untreated cells (Figure 5). The up-regulation of the ADHs and increased ethanol abundance may arise from the increased use of mixed acid fermentation for energy production in the presence of AA stress. This is further supported by the up-regulation of gene clusters associated with carbon-source utilization, including those for mannose (SPD_0295-7; > 2-fold up-regulated) and galactose (SPD_1613-16; > 4-fold) (Figure 3 and Table S2).

Genes that were differentially transcribed upon AA treatment, but not directly linked to fatty acid/membrane biology or oxidative stress tolerance included a putative bacteriocin transport system (SPD_0113-5; ~10-fold down), a putative hemolysin (SPD_1295, 3.8-fold down) and a cluster encoding a GntR-type regulator and a putative antimicrobial peptide ABC transporter (SPD_1524-6; ~5-fold down) (Figure 3). Both bacteriocins and hemolysins are known colonization and virulence factors (Chen et al., 2004; Dawid et al., 2007), hence their down-regulation upon exposure to AA may restrict the ability of the pneumococcus to cause disease. SPD_1524 encodes a YtrA-like regulator of the GntR family of transcriptional regulators, which are commonly co-transcribed with ABC transporters (Suvorova et al., 2015), as seen with this particular cluster. Although the ligands of YtrA-regulators are largely unknown, fatty acids have previously been shown to be exported by these types of ABC transporters (Suvorova et al., 2015). A putative YtrA-binding site identified upstream of SPD_1524 indicates its auto-regulation (data not shown). Further genomic analyses revealed highly homologous YtrA-binding motifs upstream of SPD_0686-8, but this cluster, encoding various efflux systems, was only marginally down-regulated (<2-fold).

Collectively, the impact of AA stress on the pneumococcal transcriptome showed that, in addition to dysregulating fatty acid biosynthesis, there was a broad range of affected genes including those of the YycFG regulon. Together, the effects mediated by AA resulted in metabolic by-products of cellular energy production suggesting that AA stress was also influencing carbon source utilization by *S. pneumoniae*.

## Conclusions

Our study is the first to examine the molecular basis of the clinically relevant LC-PUFA AA and its antimicrobial potential. Our analyses show that AA abundance increased in blood plasma upon infection, which may have substantial effects on bacteremic pathogens such as the pneumococcus. Further, we revealed a contribution by AA in killing *S. pneumoniae* within phagocytic cells, highlighting the potential significance of AA in host niches. Our subsequent analyses of the effect of AA on the pneumococcus showed that this exogenous fatty acid disrupts bacterial membrane homeostasis through two distinct pathways. First, AA is readily incorporated into bacterial membranes, which has detrimental effects on membrane integrity due to the length and number of double bonds in AA, by comparison with the endogenous membrane fatty acids of the pneumococcal membrane. Indeed, treatment with AA resulted in a significant increase in pneumococcal membrane fluidity. Intriguingly, the AA-treated cells did not display increased susceptibility to a range of different exogenous stresses, e.g., oxidative, metal or antibiotic stress. In fact, AA-treated cells were significantly more resistant to killing by the human antimicrobial peptide LL-37. The differences in membrane fluidity may also affect the correct insertion and stabilization of membrane proteins for cellular function, however this requires further investigation. The alternative mechanism of AA's antimicrobial action is achieved by compromising the pneumococcal membrane through dysregulation of the fatty acid biosynthesis genes.

Interestingly, in many other bacterial pathogens, efflux systems are upregulated in response to fatty acid toxicity (Lee and Shafer, 1999; Schielke et al., 2010). However, despite the broad impact of AA on the pneumococcal transcriptome, we did not identify any apparent resistance mechanisms that may mitigate the toxicity of AA upon this bacterium, which highlights the potential of AA as an effective anti-pneumococcal treatment strategy.

## Author contributions

BE, CT, MG, and CM designed the study. BE, SB, VP, CT, MG, and JW performed the experiments. BE, VP, SB, JP, CT, and CM wrote the manuscript. All authors have approved the final manuscript.

### Conflict of interest statement

The authors declare that the research was conducted in the absence of any commercial or financial relationships that could be construed as a potential conflict of interest.

## Supplementary material

Figure S1 The effect of AA treatment upon tolerance to antimicrobial stresses. Examination of the pneumococcal growth with or without 62.5 μM AA and with or without **(A)** 4 μg.mL^−1^ gentamicin, **(B)** 1 μg.mL^−1^ chloramphenicol, **(C)** 250 μM zinc, or **(D)** 500 μM copper. The OD_600_ was determined every 30 min. Data is representative of at least biological triplicate (± SEM) and where error bars are not visible this is due to occlusion by the shown symbols.

Figure S2 Validation of the RNA sequencing data by qRT-PCR. The mRNA transcription levels of SPD_0378, SPD_0382, SPD_0380, SPD_0309 and SPD_0772, were determined by qRT-PCR and plotted against the data obtained by RNA sequencing. Transcription levels were examined in the presence of 62.5 μM AA and corrected to untreated cells following internal normalization to *16S*. qRT-PCR data are representative of at least biological triplicates. The R^2^ value is 0.9847, which was calculated using Prism 7 (GraphPad).

Figure S3 The role of *fakB* in host fatty acid resistance. Examination of wild-type and *fakB* mutant (Δ*fakB*) growth with or without 62.5 μM AA **(A)** or DHA **(B)**. The OD_600_ was determined every 30 min. Data is representative of at least biological triplicate (±SEM) and where error bars are not visible this is due to occlusion by the shown symbols.

Figure S4 Whole cell metal content analysis of untreated and AA-treated pneumococci. *In vitro* accumulation of manganese, iron, zinc and copper were assessed via growth in C+Y with or without supplementation with 62.5 μM AA. Metal content is expressed as μg of metal per g of dry cells, as determined by ICP-MS. Data are the mean (±SEM) of at least biological triplicates. Statistical significance was determined using a two-tailed unpaired Student's *t*-test, where “ns” represents not significant.

Figure S5 The effect of AA treatment upon tolerance to oxidative stress. Examination of the pneumococcal growth with or without 62.5 μM AA and with or without **(A)** 250 μM H_2_O_2_ or **(B)** 250 μM paraquat. The OD_600_ was determined every 30 min. Data is representative of at least biological triplicate (± SEM) and where error bars are not visible this is due to occlusion by the shown symbols.

Table S1 Oligonucleotides used in this study.

Table S2 Transcriptomic data (>2-fold differential expression).

## Abbreviations

AA: arachidonic acid
PBS: phosphate-buffered saline.
