# Supplementary figures and images for: Arachidonic Acid Stress Impacts Pneumococcal Fatty Acid Homeostasis
Source: Front Microbiol. 2018 May 11;9:813. doi: 10.3389/fmicb.2018.00813 (PMC5958418; doi:10.3389/fmicb.2018.00813)

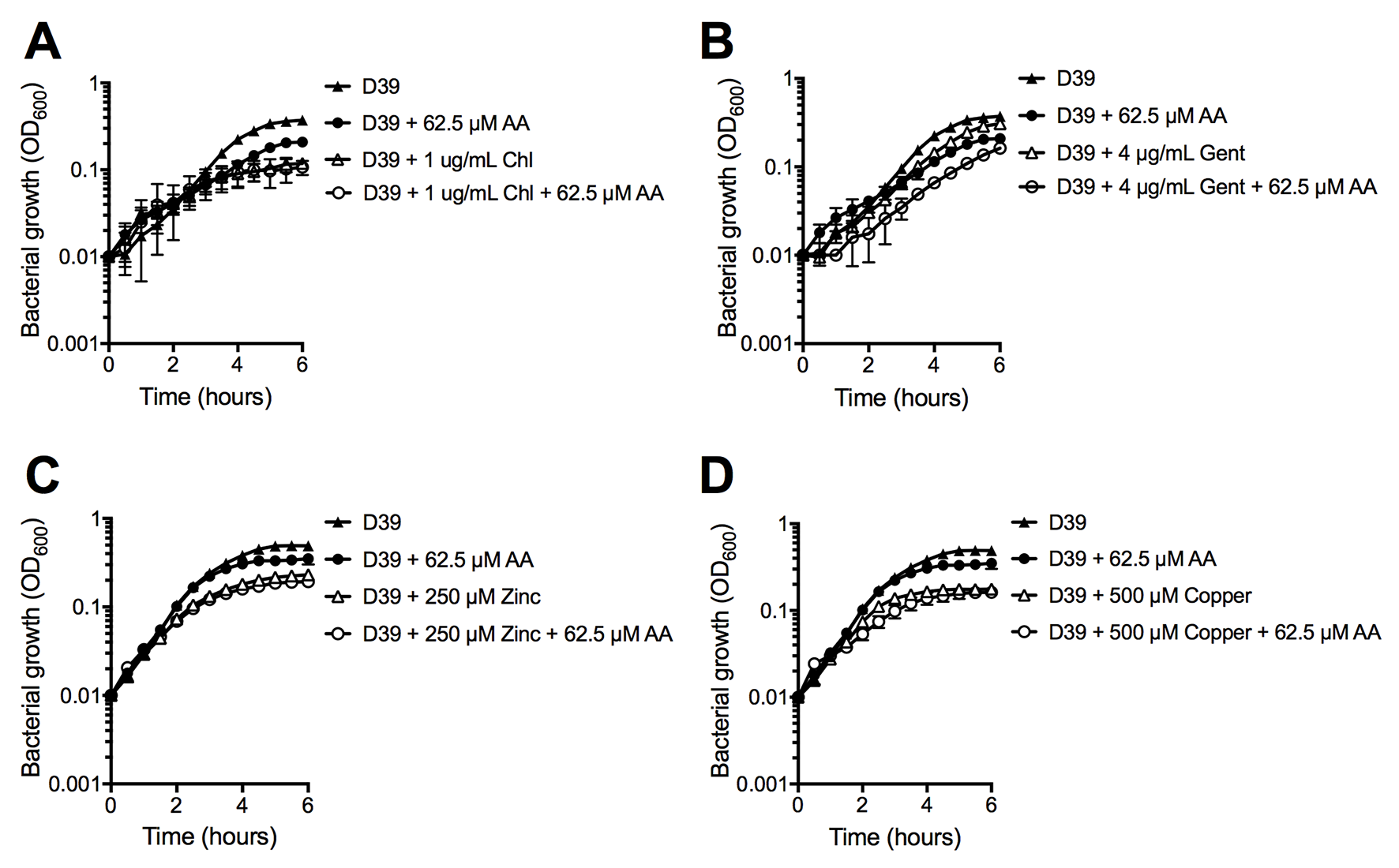

Supplement: Figure S1 — The effect of AA treatment upon tolerance to antimicrobial stresses. Examination of the pneumococcal growth with or without 62.5 μM AA and with or without (A) 4 μg.mL−1 gentamicin, (B) 1 μg.mL−1 chloramphenicol, (C) 250 μM zinc, or (D) 500 μM copper. The OD600 was determined every 30 min. Data is representative of at least biological triplicate (± SEM) and where error bars are not visible this is due to occlusion by the shown symbols. [file Image_1.tiff]

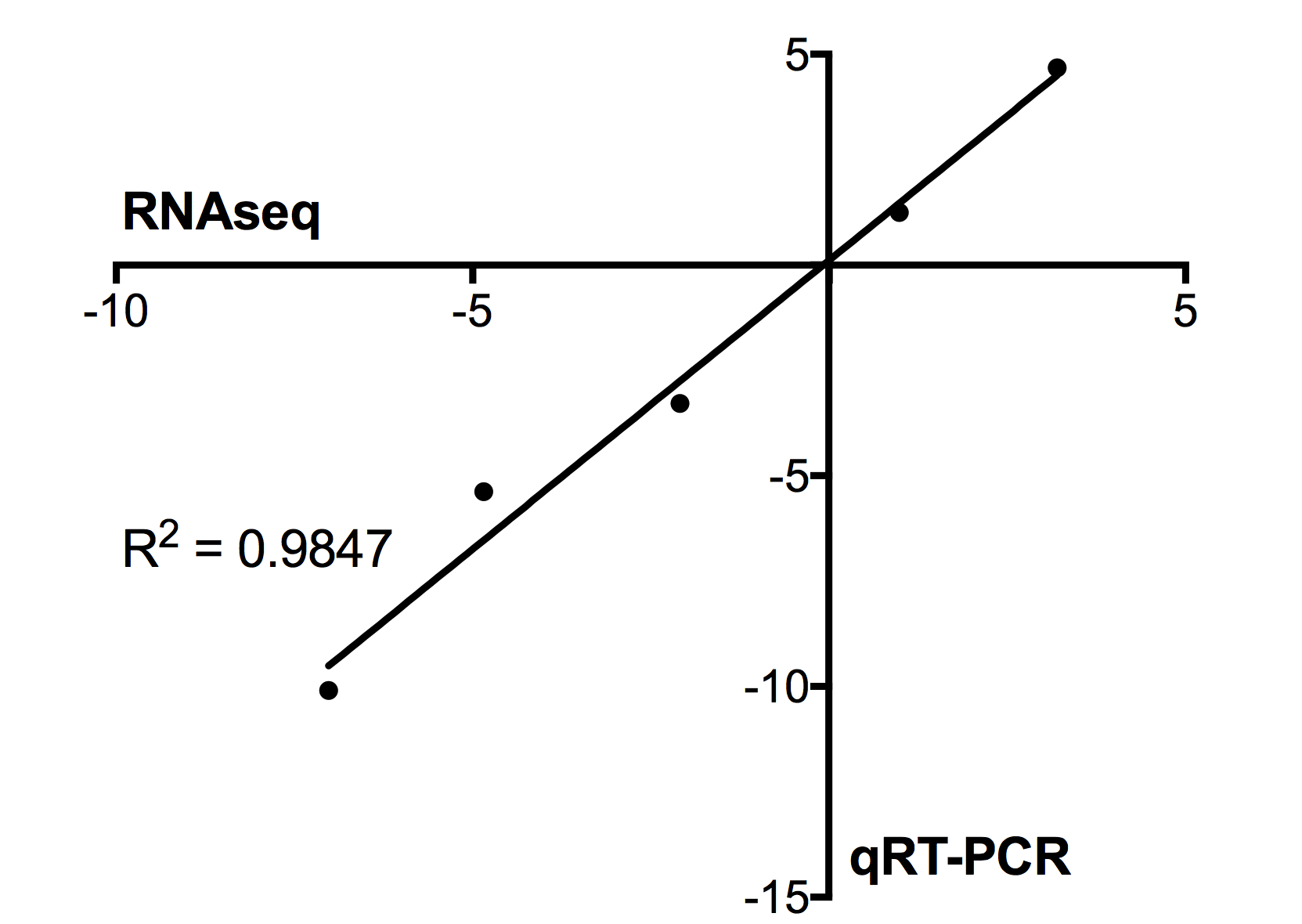

Supplement: Figure S2 — Validation of the RNA sequencing data by qRT-PCR. The mRNA transcription levels of SPD_0378, SPD_0382, SPD_0380, SPD_0309 and SPD_0772, were determined by qRT-PCR and plotted against the data obtained by RNA sequencing. Transcription levels were examined in the presence of 62.5 μM AA and corrected to untreated cells following internal normalization to 16S. qRT-PCR data are representative of at least biological triplicates. The R2 value is 0.9847, which was calculated using Prism 7 (GraphPad). [file Image_2.TIFF]

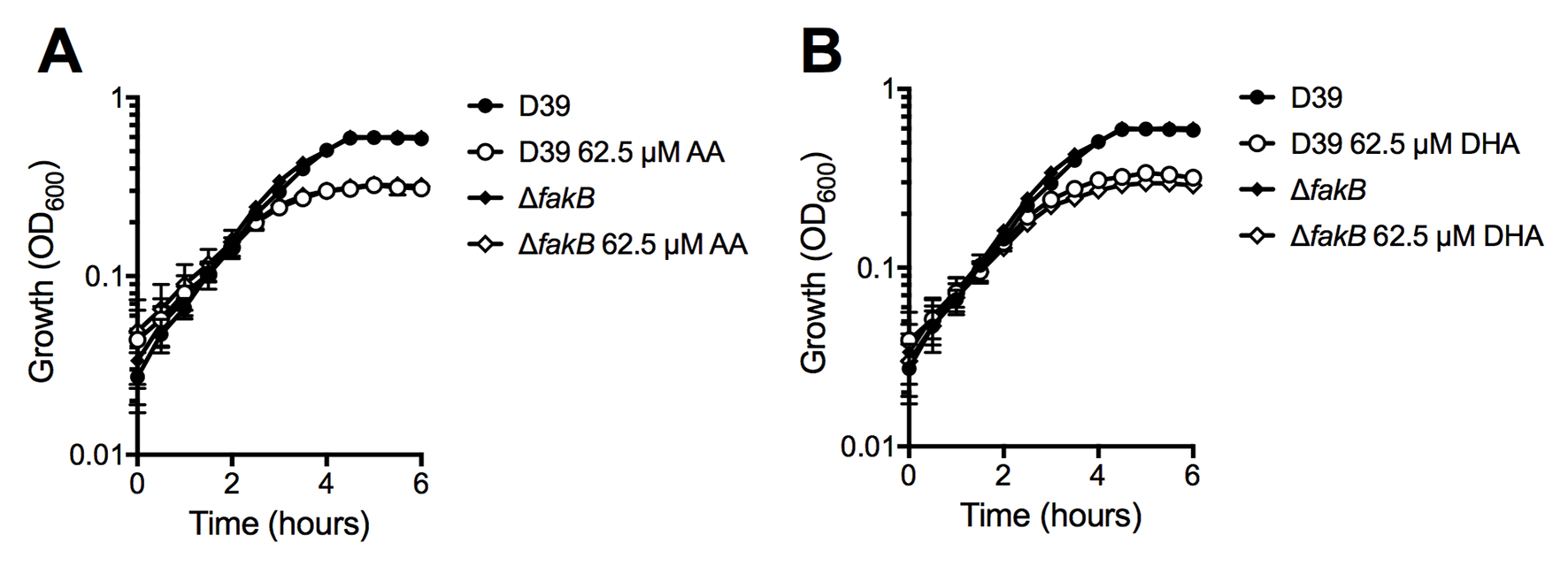

Supplement: Figure S3 — The role of fakB in host fatty acid resistance. Examination of wild-type and fakB mutant (ΔfakB) growth with or without 62.5 μM AA (A) or DHA (B). The OD600 was determined every 30 min. Data is representative of at least biological triplicate (±SEM) and where error bars are not visible this is due to occlusion by the shown symbols. [file Image_3.TIFF]

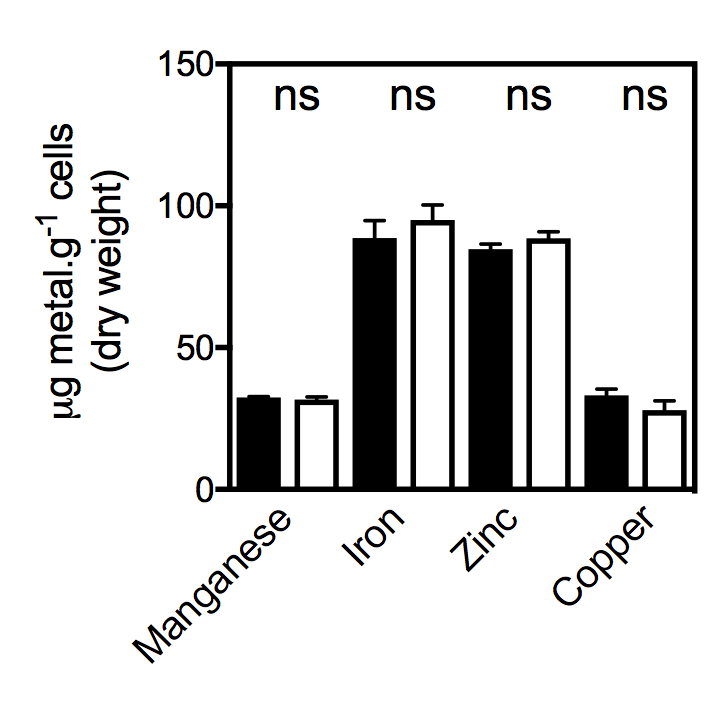

Supplement: Figure S4 — Whole cell metal content analysis of untreated and AA-treated pneumococci. In vitro accumulation of manganese, iron, zinc and copper were assessed via growth in C+Y with or without supplementation with 62.5 μM AA. Metal content is expressed as μg of metal per g of dry cells, as determined by ICP-MS. Data are the mean (±SEM) of at least biological triplicates. Statistical significance was determined using a two-tailed unpaired Student's t-test, where “ns” represents not significant. [file Image_4.TIFF]

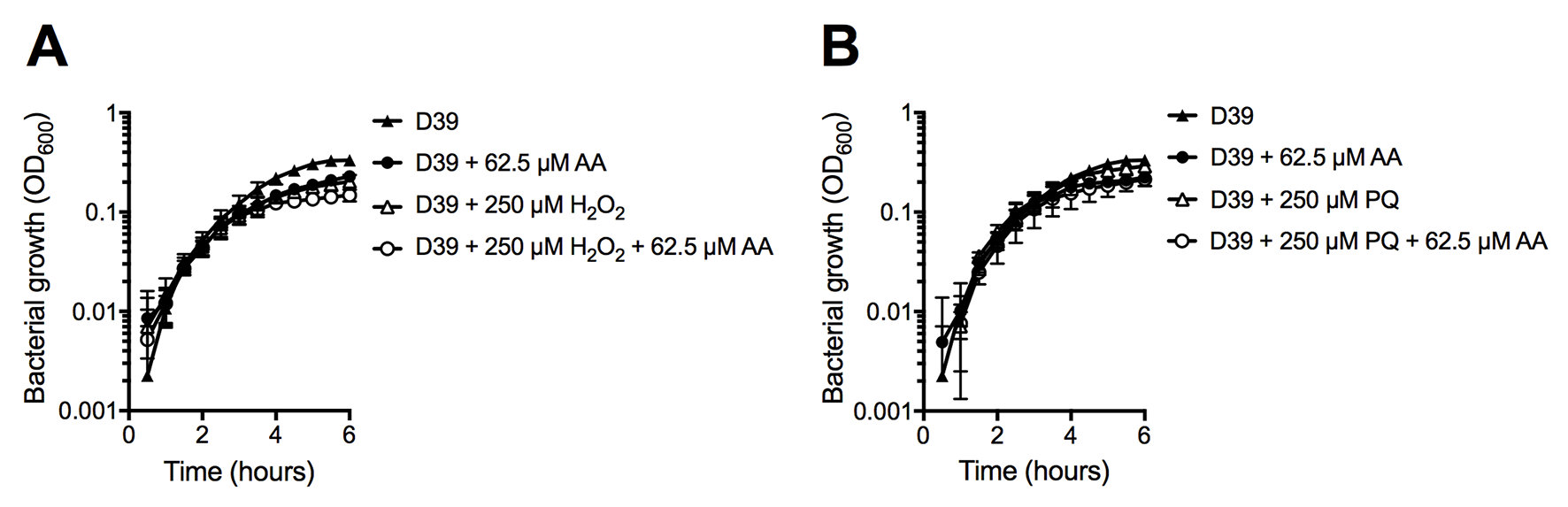

Supplement: Figure S5 — The effect of AA treatment upon tolerance to oxidative stress. Examination of the pneumococcal growth with or without 62.5 μM AA and with or without (A) 250 μM H2O2 or (B) 250 μM paraquat. The OD600 was determined every 30 min. Data is representative of at least biological triplicate (± SEM) and where error bars are not visible this is due to occlusion by the shown symbols. [file Image_5.TIFF]
