# Supplementary material for: Arachidonic Acid Stress Impacts Pneumococcal Fatty Acid Homeostasis
Source: Front Microbiol. 2018 May 11;9:813. doi: 10.3389/fmicb.2018.00813 (PMC5958418; doi:10.3389/fmicb.2018.00813)
Supplement: Table S1 — Oligonucleotides used in this study. [file Table_1.docx]

| **Primer** | **Sequence (5' - 3')** |
| --- | --- |
| qD39_16S_F | CATGCAAGTAGAACGCTGAA |
| qD39_16S_R | TGTCATGCAACATCCACTCT |
| qSPD_0309_F | TGGACCAGCCCTAGCCTTTGAA |
| qSPD_0309_R | CACACTTGACTAAGGAAGAC |
| qSPD_0378_F | CTGAGGTCGCAAATGGTTTT |
| qSPD_0378_R | CCCGCTTCATCTCTACCAAA |
| qSPD_0380_F | CAGATTCGATGATGCCCTCT |
| qSPD_0380_R | AAGCGACCAGAAGCGATAAA |
| qSPD_0382_F | GGCTGGCTTTATGCTAGGTG |
| qSPD_0382_R | CGAACAGCATGACCAAAGTG |
| qSPD_0772_F | TAGGCAATGTTATCTATAAG |
| qSPD_0772_R | TTGGCAAAGCCTCTACTGAA |
| delSPD_0646 up | TCCTGCATGGCTTGGACAAA |
| delSPD_0646 low ery | TTGTTCATGTAATCACTCCTTCGCAATAATCTTCCAAGTCAT |
| delSPD_0646 up ery | CGGGAGGAAATAATTCTATGAGATGGGCTATGAAATTGATTAA |
| delSPD_0646 low | GGACAAACTGGTCATCTAC |
