# Supplementary material for: Arachidonic Acid Stress Impacts Pneumococcal Fatty Acid Homeostasis
Source: Front Microbiol. 2018 May 11;9:813. doi: 10.3389/fmicb.2018.00813 (PMC5958418; doi:10.3389/fmicb.2018.00813)
Supplement: Table S2 — Transcriptomic data (>2-fold differential expression). [file Table_2.docx]

| **Locus Tag** | **Gene Name** | **Fold-Change** | **Protein name/function** |
| --- | --- | --- | --- |
| SPD_0166 | *ribH* | 15.1 | 6,7-dimethyl-8-ribityllumazine synthase |
| SPD_0167 | *ribB* | 14.5 | Riboflavin biosynthesis protein RibBA |
| SPD_0168 | *ribE* | 13.7 | Riboflavin synthase, alpha subunit |
| SPD_0169 | *ribD* | 12.2 | Riboflavin biosynthesis protein RibD |
| SPD_0373 | *ahpD* | 5.5 | Alkyl hydroperoxide reductase AhpD |
| SPD_0458 | *hrcA* | 5.6 | Heat-inducible transcription repressor HrcA |
| SPD_0459 | *grpE* | 5.8 | Protein GrpE (HSP-70 cofactor) |
| SPD_0460 | *dnaK* | 5.4 | Chaperone protein DnaK (HSP70) (Heat shock 70 kDa protein) (Heat shock protein 70) |
| SPD_0461 | *dnaJ* | 3.2 | Chaperone protein DnaJ |
| SPD_0771 | *lacR1* | 3.5 | Lactose phosphotransferase system repressor |
| SPD_0772 |  | 3.2 | Tagatose-6-phosphate kinase (EC 2.7.1.144) |
| SPD_0773 | *fruA* | 3.4 | PTS system, fructose specific IIABC components (EC 2.7.1.69) |
| SPD_1461 | *psaB* | 2.9 | Manganese ABC transporter, ATP-binding protein |
| SPD_1462 | *psaC* | 2.9 | Manganese ABC transporter, permease protein |
| SPD_1463 | *psaA* | 3.4 | Manganese ABC transporter, substrate binding protein |
| SPD_1606 |  | 3.1 | MgtC/SapB family protein |
| SPD_1607 |  | 3.7 | ABC transporter, permease protein |
| SPD_1608 |  | 3.5 | ABC transporter ATP-binding protein |
| SPD_1609 |  | 3.8 | ABC transporter, substrate-binding protein |
| SPD_1610 |  | 3.9 | Uncharacterized protein |
| SPD_1611 |  | 3.0 | Uncharacterized protein |
| SPD_1612 | *galE-2* | 3.2 | UDP-glucose 4-epimerase (EC 5.1.3.2) |
| SPD_1613 | *galT-1* | 4.0 | Galactose-1-phosphate uridylyltransferase (Gal-1-P uridylyltransferase) (EC 2.7.7.12) (UDP-glucose--hexose-1-phosphate uridylyltransferase) |
| SPD_1834 | *adhE* | 4.4 | Iron-containing Aldehyde-alcohol dehydrogenase |
| SPD_1865 |  | 4.7 | Alcohol dehydrogenase, zinc-containing |
| SPD_1899 |  | 4.7 | Glutamine amidotransferase, class 1 |
| SPD_2037 | *cysK* | 4.3 | Cysteine synthase (EC 2.5.1.47) |
| SPD_2059 |  | 7.1 | Phage infection protein |
| SPD_0113 |  | -9.0 | pseudogene |
| SPD_0114 |  | -11.3 | Uncharacterized protein |
| SPD_0115 |  | -12.3 | Orphan ATP-binding protein |
| SPD_0116 |  | -10.1 | Uncharacterized protein |
| SPD_0117 |  | -5.4 | Uncharacterized protein |
| SPD_0118 |  | -4.0 | Uncharacterized protein |
| SPD_0119 |  | -4.0 | Membrane protein, putative |
| SPD_0120 |  | -3.9 | Membrane protein, putative |
| SPD_0121 |  | -3.5 | Uncharacterized protein |
| SPD_0122 |  | -3.3 | Uncharacterized protein |
| SPD_0123 |  | -2.5 | Uncharacterized protein |
| SPD_0124 |  | -2.7 | Membrane protein, putative |
| SPD_0378 | *fabM* | -7.0 | Enoyl-CoA hydratase/isomerase family protein |
| SPD_0380 | *fabH* | -2.1 | 3-oxoacyl-[acyl-carrier-protein] synthase 3 (EC 2.3.1.180) (3-oxoacyl-[acyl-carrier-protein] synthase III) (Beta-ketoacyl-ACP synthase III) (KAS III) |
| SPD_0382 | *fabK* | -4.8 | Trans-2-enoyl-ACP reductase II |
| SPD_0383 | *fabD* | -4.7 | Malonyl CoA-acyl carrier protein transacylase (EC 2.3.1.39) |
| SPD_0384 | *fabG* | -4.5 | 3-oxoacyl-(Acyl-carrier-protein) reductase (EC 1.1.1.100) |
| SPD_0385 | *fabF* | -3.9 | 3-oxoacyl-[acyl-carrier-protein] synthase 2 (EC 2.3.1.179) |
| SPD_0386 | *accB* | -3.9 | Acetyl-CoA carboxylase, biotin carboxyl carrier protein |
| SPD_0387 | *fabZ* | -4.0 |  |
| SPD_0388 | *accC* | -3.9 | Acetyl-CoA carboxylase, biotin carboxylase (EC 6.4.1.2) |
| SPD_0389 | *accD* | -3.7 |  |
| SPD_0390 | *accA* | -3.6 |  |
| SPD_0391 | SPD_0391 | -4.7 | Uncharacterized protein |
| SPD_0392 | SPD_0392 | -3.9 | Uncharacterized protein |
| SPD_0646 | SPD_0646 | -3.7 | Uncharacterized protein |
| SPD_0684 | SPD_0684 | -4.5 | Biotin synthase (BioY family protein) |
| SPD_0775 | SPD_0775 | -4.7 | Uncharacterized protein |
| SPD_0913 | SPD_0913 | -9.2 | Uncharacterized protein |
| SPD_1294 | SPD_1294 | -3.6 | Uncharacterized protein |
| SPD_1295 | SPD_1295 | -3.8 | Hemolysin |
| SPD_1524 | SPD_1524 | -5.5 | Transcriptional regulator, GntR family protein |
| SPD_1525 | SPD_1525 | -5.0 | Two-component associated ABC transporter (antimicrobial peptide), ATP-binding protein, putative |
| SPD_1526 | SPD_1526 | -4.4 | Two-component associated ABC transporter (antimicrobial peptide), permease protein, putative |
| SPD_1874 | SPD_1874 | -4.5 | LysM domain protein |
| SPD_2068 | *htrA/degP* | -5.6 | Serine protease; htrA; serine protease; K04771 serine protease Do [EC:3.4.21.107] |
| SPD_2069 | *spoJ* | -4.4 | SpoJ protein |
